# Evaluation of a Simple Low-cost Intervention to Empower People with CKD to Reduce Their Dietary Salt Intake: OxCKD1, a Multicenter Randomized Controlled Trial

**DOI:** 10.34067/KID.0000000000000160

**Published:** 2023-05-31

**Authors:** Christopher A. O'Callaghan, Clare Camidge, Rachel Thomas, Michael E. Reschen, Alison J. Maycock, Daniel S. Lasserson, Robin A. Fox, Nicholas P.B. Thomas, Brian Shine, Tim James

**Affiliations:** 1 Nuffield Department of Medicine, University of Oxford, Oxford, United Kingdom; 2 Oxford University Hospitals NHS Foundation Trust, Oxford, United Kingdom; 3 Dietetics Department, Oxford University Hospitals NHS Foundation Trust, Oxford, United Kingdom; 4 Hollow Way Medical Centre, Ivy Close, Oxford, United Kingdom; 5 Division of Health Sciences, Warwick Medical School, University of Warwick, Coventry, United Kingdom; 6 Bicester Health Centre, Coker Close, Bicester, Oxfordshire, United Kingdom; 7 Windrush Medical Centre, Welch Way, Witney, Oxfordshire, United Kingdom; 8 Department of Clinical Biochemistry, Oxford University Hospitals NHS Foundation Trust, Oxford, United Kingdom

**Keywords:** CKD, CKD, chronic renal insufficiency, clinical nephrology, clinical trial, patient-centered care, randomized controlled trials

## Abstract

**Key Points:**

- A randomized controlled trial demonstrates that a simple and cheap 1-month intervention empowers people with CKD to lower their dietary salt intake.
- The effect of the intervention persisted after the intervention finished.

**Background:**

To evaluate the efficacy of a simple low-cost intervention to empower people with CKD to reduce their dietary salt intake.

**Methods:**

A randomized controlled trial in primary and secondary care comparing the OxSalt care bundle intervention versus standard care for 1 month. Participants were people with CKD and an eGFR >20 ml/min per 1.73 m^2^ and were recruited from primary and secondary care. The primary outcome was a reduction in dietary salt intake, as assessed by 24-hour urinary sodium excretion, after 1 month of the intervention.

**Results:**

Two hundred and one participants were recruited. Dietary salt intake, as assessed from 24-hour urine sodium excretion, fell by 1.9 (±2.9) g/d in the intervention group compared with 0.4 (±2.7) g/d in the control group (*P* < 0.001). Salt intake was still reduced to a lesser extent over the following year in the intervention group.

**Conclusions:**

A short, low-cost, easily delivered intervention empowers people with CKD to reduce their dietary salt intake.

**Trial registration:**

ClinicalTrials.gov NCT01552317.

## Graphical abstract

**Figure:**
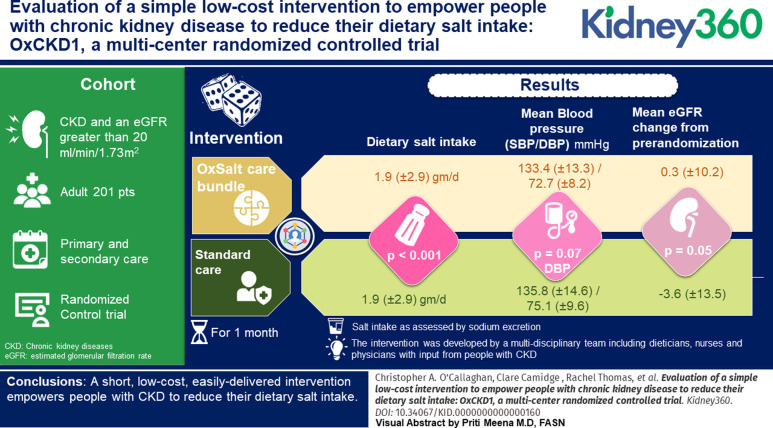

## Introduction

Increased dietary salt intake is associated with high BP, cardiovascular risk, and mortality.^1–3^ However, salt continues to be widely used in domestic and commercial food preparation, and consumption of salt remains high in almost all countries, globally averaging over 15 g of salt per day.^4^ It was estimated that high salt intake was responsible for approximately 3 million excess deaths globally in 2017 alone and 70 million disability-adjusted life-years.^4^ Compared with other dietary factors, salt ranked top for its effect on mortality in men and was the leading dietary risk factor for both deaths and disability-adjusted life-years in East Asia, the high-income Asia pacific regions, China, Japan and Thailand. The mean UK salt intake was recently estimated at 8.4 g/d.^5^

There is a consensus that elevated salt intake is associated with increased BP and that this relationship is enhanced in people with hypertension.^6,7^ A meta-analysis of 103 trials of salt reduction demonstrated an average decrease in systolic BP of 3.8 mm Hg for every 5.8 g reduction in salt intake, which rose by age, such that there was a decrease, respectively, of 5.84 mm Hg for people of age 70 years and a further decrease of 1.87 mm Hg in people with hypertension.^8^ For the general population, the World Health Organization has recommended a salt intake of no more than 5 g/d.^9^

A 2021 Cochrane review and meta-analysis identified 21 studies in CKD and concluded that there was high-certainty evidence that lowering salt intake reduced both systolic and diastolic BP and albuminuria.^10^ Data from the Chronic Renal Insufficiency Cohort study demonstrate clear associations of salt intake with cardiovascular risk^11^ and progression of CKD.^12^ The addition of moderate salt restriction to an angiotensin-converting enzyme inhibitor in patients with nondiabetic CKD (target salt intake 2.9 g/d, achieved 6.2 g/d) reduced proteinuria and BP more than the addition of an angiotensin receptor blocker.^13^

Clinical trials designed to evaluate the benefit of lowering salt intake in CKD have deployed a range of intensive, personalized, and relatively expensive interventions to drive down salt intake during the study period.^10^ These intensive interventions have included personalized dietary advice and counseling,^13^ motivational coaching,^14^ cooking lessons,^15^ and feedback from monitoring of 24-hour urine sodium excretion.^16^ Unfortunately, these intensive personalized interventions are not feasible or affordable in most health care systems, which are faced with very large numbers of people with CKD, so such interventions cannot be provided as part of routine clinical care.

Kidney Disease: Improving Global Outcomes recommends a dietary salt intake of <5 g/d unless contraindicated.^17^ National guidelines generally recommend dietary salt reduction in CKD, and in the United Kingdom, the National Institute for Health and Care Excellence (NICE) recommends offering dietary advice about salt intake appropriate to the severity of the CKD.^18^ Nevertheless, targets for salt reduction can be the least achieved management targets for people with CKD.^19,20^

A clinician or person with CKD reviewing the available evidence and guidance could reasonably conclude that a reduction in salt intake is of benefit in CKD. However, it remains unclear how this recommendation can be converted into a real reduction in salt intake because there is a paucity of evidence about feasible and affordable interventions to help individual people with CKD achieve the salt reductions that are recommended. Therefore, it remains challenging for people to reduce their salt intake, especially if processed food is a major element of their diet.

To address this evidence gap, we undertook a randomized controlled trial to test whether a simple low-cost intervention could help to empower people with CKD to reduce their dietary salt intake.

## Methods

### Trial Design

**Figure 1:**
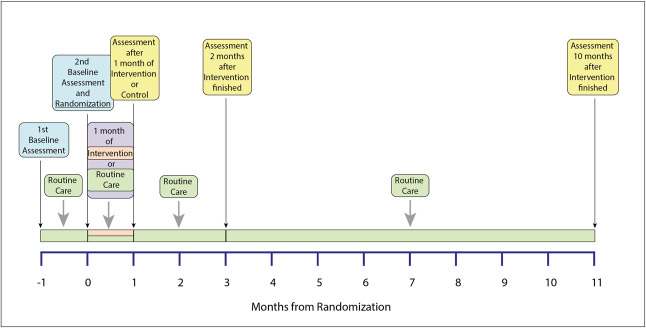
**Flowchart of the study**.

The trial was a randomized controlled trial with the design shown in Figure 1. After a 1-month run-in period, participants were randomized to 1 month of the intervention or 1 month of normal routine care. After this, all participants resumed normal routine care. There were no substantive changes to the methods after the trial commencement. Participants received reasonable travel expenses, but no other remuneration.

### Participants

People 18 years or older with CKD with an eGFR >20 ml/min per 1.73 m^2^ were identified using electronic medical record searches and invited to participate through letter. Recruitment was undertaken in primary and secondary care. To be eligible for inclusion, potential participants had to be 18 years or older with capacity to give informed consent, have a diagnosis of CKD with an eGFR >20 ml/min per 1.73 m^2^, and be able to comply with the study requirements. The 2009 Chronic Kidney Disease Epidemiology Collaboration equation was used to calculate eGFRs for recruitment, and eGFR values were corrected for ethnicity in line with guidance at the time of recruitment. Exclusion criteria included any condition, such as a salt-losing nephropathy, that would put the patient at risk from the intervention; an inability to participate in the study, including an inability to understand English or special communication needs (the intervention used English and funds were not available to provide other forms of communication or other languages); and women who were pregnant, breast feeding, or planning a pregnancy. There was no formal or other requirement for participants to have a computer, smartphone, or internet access. Ethnicity was self-reported according to the UK National Health Service coding system and in line with monitoring of inclusive recruitment to clinical trials. We anticipated a daily sodium excretion of around 180 mmol/d in the control group and allowed for a wide standard deviation of 80 mmol/d. For the desired power of 0.8 at the 0.05 significance with a standard deviation of 80 mmol/d, a sample size of at least 63 participants is required in each group to detect a reduction in sodium excretion from 180 to 140 mmol/d (and at least 82 participants to detect a reduction to 145 mmol/d). We, therefore, proposed to recruit 100 participants in each group to allow for participants drop-out. This study was undertaken in adherence to the Declaration of Helsinki.

Recruitment and follow-up were undertaken in centers in primary and secondary care, including two hospital clinics (Oxford University Hospitals and Milton Keynes University Hospital) and four general practice health centers (Hollow Way Medical Center, Oxford; Windrush Medical Center, Witney; Bicester Health Center, Bicester; and Church Street Practice, Wantage). Recruitment took place between August 6, 2012, and May 22, 2014. Recruitment was stopped when the number of participants exceeded the target of 200. Eight participants consented to participate in this study but did not attend for randomization after 1 month of normal routine care nor for further study visits. Two assessments of 24-hour urine sodium content were taken, and their average used as a baseline against which values from multiple further time points were compared.

### Interventions

The intervention was the OxSalt care bundle which is described further in the Supplemental Material. The intervention was developed by a multidisciplinary team, including dieticians, nurses, and physicians with input from people with CKD. The intervention was based on three principles to empower people with CKD: to understand the health benefits of reducing salt intake, to understand how to evaluate the salt content of food, and to understand how to select or prepare food that is appetizing and low in salt content. The intervention included an initial digital briefing, information in electronic and written form, online resources, and real-time electronic reminders.

### Outcomes

The primary outcome measure was a reduction in daily dietary salt intake at 1 month, assessed by measuring 24-hour urine sodium excretion. The secondary outcome measures included changes in salt intake at 3 and 11 months postrandomization (as assessed by measuring 24-hour urine sodium excretion) and BP and kidney function at 1, 3, and 11 months post-randomization (as assessed by blood tests taken at these study visits). For measurement of BPs, all participants were provided with fully automated validated BP monitors (Omron, Kyoto, Japan) to take home and provided with instruction on their use in accordance with UK guidance from NICE.^21^

### Randomization

This was undertaken using the OxMaR software package^22^ which allowed for remote web-based automated electronic randomization with minimization on the basis of age, sex, ethnicity, and whether the person had diabetes. Laboratory analysis of samples and clinical care of participants were undertaken by staff who were blinded to the randomization status of the participants. Urine sodium was analyzed using an ion-selective electrode and urine and plasma creatinine using enzymatic analysis, both on the Abbott Architect C8000 analyzer (Abbott Laboratories, Maidenhead, UK). Both methods demonstrated good longitudinal assay accuracy through participation in external quality assurance programs.

### Analytical Methods

Statistical analyses were undertaken using R version 4.2.2.^23^ For all analyses, eGFRs for all time points were calculated using the 2009 Chronic Kidney Disease Epidemiology Collaboration equation, and in accordance with current guidance, eGFR values were not corrected for ethnicity.^18^ Comparisons were conducted as indicated in the relevant Results section, Tables, or Figures. *P* values for comparisons between the control and treatment groups were calculated using a chi-squared test, Fisher exact test, Welch *t* test, or *t* test as appropriate to the data type and distribution. For mixed-effect models, residual plots did not reveal any obvious deviations from homoscedasticity or normality, and *P* values were obtained by likelihood ratio tests of the full model with the effect in question against the model without the effect in question.

### Registration

The study was registered on March 1, 2012, with ClinicalTrials.gov, Registration Code: NCT01552317.

## Results

### Recruitment and Follow-up

People who fulfilled the eligibility criteria were invited to participate through primary and secondary care centers. Recruitment was undertaken on a first-come-first-served basis across multiple centers (583 invited, 201 recruited, 81 declined, 22 were ineligible on further assessment, and 279 were not recruited nor assessed further as the recruitment target was reached). Following informed consent, there was a 1-month period of normal routine care after which participants were randomized to the intervention or to normal routine care (the control group). All participants were blinded to the nature of the intervention (see Supplemental Material) before randomization.

A total of 201 participants consented to take part in this study, of whom 193 attended for randomization and were randomized. Ninety-seven participants were randomized to the control group, and 96 participants were randomized to the intervention group. The mean age of all participants was 63.7 (±11.5) years, and there was no statistically significant difference in the mean ages of the control and intervention groups, nor in their educational attainment nor use of key medications (Table 1 and Supplemental Tables 1 and 2). The baseline characteristics of the participants who were randomized to each group are presented in Table 1. The scheme for this study is illustrated in Figure 1, and both baseline assessments took place before randomization. The intervention lasted for 1 month, and all participants resumed normal routine care after this. No adverse effects of the intervention were identified.

**Table 1:** Baseline characteristics of participants

Characteristic	Group		
	Control, *N*=97^a^	Intervention, *N*=96^a^	*P* Value^b^
**Sex**			0.8
Female	48/97 (49%)	49/96 (51%)	
Male	49/97 (51%)	47/96 (49%)	
Age	64.2 (10.5)	63.3 (12.8)	0.6
Diabetes	25/97 (26%)	22/96 (23%)	0.6
**Ethnicity**			0.6
African	0/97 (0%)	2/96 (2.1%)	
Any other Asian background	0/97 (0%)	1/96 (1.0%)	
Any other ethnic group	1/97 (1.0%)	0/96 (0%)	
Any other mixed background	1/97 (1.0%)	1/96 (1.0%)	
Any other White background	3/97 (3.1%)	5/96 (5.2%)	
British	86/97 (89%)	84/96 (88%)	
Caribbean	2/97 (2.1%)	0/96 (0%)	
Irish	2/97 (2.1%)	2/96 (2.1%)	
Pakistani	0/97 (0%)	1/96 (1.0%)	
White and Asian	1/97 (1.0%)	0/96 (0%)	
White and Black African	1/97 (1.0%)	0/96 (0%)	
**Smoking status**			>0.9
Current	10/97 (10%)	9/96 (9.4%)	
Never	46/97 (47%)	48/96 (50%)	
Previously	41/97 (42%)	39/96 (41%)	
Hypertension	65/97 (67%)	61/96 (64%)	0.6
Body mass index	29.1 (5.6)	30.2 (6.5)	0.2
eGFR	68.7 (34.4)	60.0 (25.8)	0.082

a *n*/*N* (%); mean (SD).

b Pearson chi-squared test; Welch Two Sample *t* test; Fisher exact test.

### Assessment of Baseline Salt Intake from 24-hour Urine Sodium Excretion

Dietary salt intake was assessed by measuring 24-hour urine sodium excretion from 24-hour urine collections. The mean first baseline urinary sodium excretion for all participants across the control and intervention groups was 130.2 (±59.7) mmol/24 hours, representing a mean salt intake of 7.6 (±3.5) g/d. Following consent, all participants continued with 1 month of normal routine care and were then randomized to either 1 month of the intervention or a further 1 month of continued normal routine care. Immediately before randomization, a second baseline measurement of sodium excretion was made. Neither the first (*P* = 1), the second (*P* = 0.1), nor the mean (*P* = 0.3) of these baseline measurements differed significantly between the control and intervention groups. The mean value of these two measurements for each participant was used as the baseline sodium excretion for subsequent analysis.

### Primary Outcome: Effect of the Intervention on Salt Intake as Assessed by Sodium Excretion

The primary outcome of this study was a reduction, after 1 month of the intervention, in dietary salt intake as assessed by 24-hour urine sodium excretion. Therefore, after randomization to 1 month of the intervention or 1 month of continued normal routine care, sodium excretion was reassessed. After 1 month of the intervention, the mean 24-hour sodium excretion was 92.9 (±48.4) mmol/24 hours for the intervention group, compared with 118.9 (±52.4) mmol/24 hours for the control group, who continued to receive normal routine care (*P* = 0.001).

**Figure 2:**
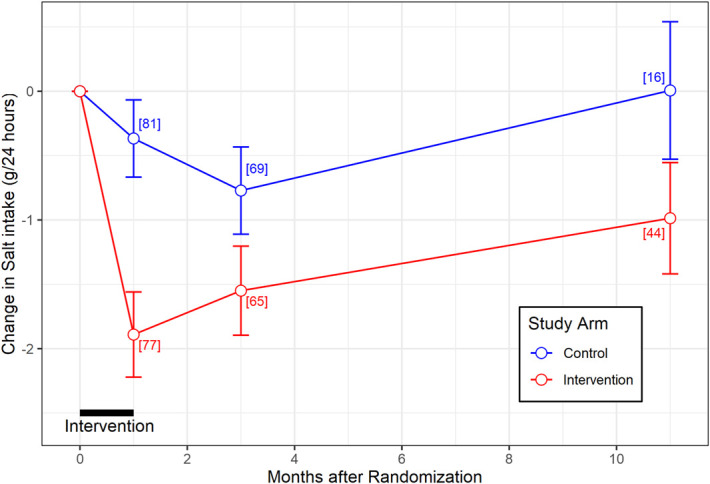
**Changes in dietary salt intake from baseline (mean±SEM).** The number of observations made at each time point after randomization is indicated in red for the intervention group and blue for the control group. Dietary salt intake per day was assessed by measuring 24-hour urine sodium excretion.

After 1 month of the intervention, the mean change in 24-our sodium excretion from baseline was −6.28 (±46.2) mmol/24 hours for the control group and −32.4 (±49.7) mmol/24 hours for the intervention group (*P* = 8×10^−4^). This represents a reduction in salt intake of −1.9 (±2.9) g for the intervention group compared with −0.4 (±2.7) g for the control group (*P* = 8×10^−4^) (Figure 2). The mean percentage change in salt intake from baseline for individual participants was −20.6 (±36.2)% for the intervention group compared with +0.7 (±46)% for the control group (*P* = 0.002).

### Trajectory of Salt Intake after the End of the Intervention as Assessed by Sodium Excretion

The intervention lasted for 1 month, and all participants resumed normal routine care after this. The trajectory of their salt intake is shown in Figure 2. For participants in the intervention group, there was a significant reduction from their baseline sodium excretion at all time points after randomization: −32.4 (±49.7) mmol/24 hours (*P* = 2×10^−7^) at the end of the 1 month intervention, −26.5 (±47.8) mmol/24 hours (*P* = 3×10^−5^) 2 months after the end of the intervention, and −16.9 (±49.1) mmol/24 hours (*P* = 0.03) 11 months after the end of the intervention (Supplemental Figure 1 and Supplemental Tables 3 and 4). For participants in the control group, this was not the case and there was not a consistent significant reduction in sodium excretion over time: −6.3 (±46.2) mmol/24 hours (*P* = 0.2) after 1 month of normal routine care during the study period, −13.2 (±48.2) mmol/24 hours (*P* = 0.03) after 2 months of further follow-up, and 0.1 (±36.6) mmol/24 hours (*P* = 1) after 11 months of further follow-up.

### Changes in Sodium:Creatinine Ratio

**Figure 3:**
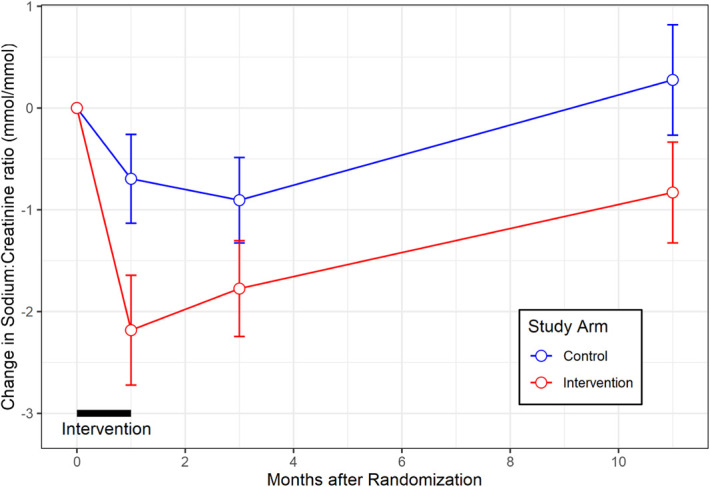
Changes in urine sodium:creatinine ratio over time (mean±SEM).

Because there is potential for human error to lead to incomplete collection of 24-hour urine output, we, therefore, analyzed the sodium:creatinine ratio in the collected urine samples (Figure 3). At baseline, the mean urine sodium:creatinine ratio was 10.6 (±4) mmol/mmol in the intervention group and 11.7 (±6.9) mmol/mmol in the control group (*P* = 0.2). After 1 month of the intervention, the mean urine sodium:creatinine ratio was 8 (±4.1) mmol/mmol for the intervention group, compared with 10.1 (±3.6) mmol/mmol for the control group, who continued to receive normal care (*P* = 6×10^−4^). Aggregating over all time points after randomization, the mean urine sodium:creatinine ratio was significantly lower in the intervention group at 8.4 (±3.5) mmol/mmol, compared with 10 (±3.7) mmol/mmol for the control group (*P* = 2×10^−5^).

There were significant differences at each subsequent time point after randomization between intervention and control groups in urine sodium:creatinine ratio. At 3 months after randomization, the urine sodium:creatinine ratio was 8.4 (±2.9) mmol/mmol for the intervention group, compared with 9.9 (±4.1) mmol/mmol for the control group (*P* = 0.009). At 11 months after randomization, the urine sodium:creatinine ratio was 8.9 (±3.4) mmol/mmol for the intervention group, compared with 10.3 (±2.3) mmol/mmol for the control group (*P* = 0.04).

### Longer-Term Trajectory of Sodium Excretion

Across the whole period after randomization, sodium intake was lower at 98.6 (±44.3) mmol/24 hours in the intervention group, compared with 116.5 (±52.7) mmol/24 hours in the control group (*P* = 7×10^−4^). In the postrandomization period, the mean reduction in sodium intake was greater at −17.6 (±41.7) mmol/24 hours in the intervention group, compared with −5.4 (±36.9) mmol/24 hours in the control group (*P* = 3×10^−4^). We undertook a linear mixed-effects analysis of the relationship between sodium excretion and study arm in the whole period after randomization. As fixed effects, we included study group, and as random effects, we included intercepts for participants and visit. The coefficient for study arm was −12 (SEM 4.1), and the inclusion of study arm to the model was highly significant (*P* = 10^−7^), demonstrating a strong influence of the intervention on salt intake.

### Effect of the Intervention on BP and Kidney Function

The mean home BPs at baseline were 133.4 (±13.3)/72.7 (±8.2) mm Hg for the intervention group and 135.8 (±14.6)/75.1 (±9.6) mm Hg for the control group (*P* = 0.3 for systolic and *P* = 0.07 for diastolic pressures, respectively). After randomization to 1 month of the intervention or 1 month of continued routine care, the mean systolic BP was 128.7 (±14.3) mm Hg for the intervention group, compared with 132.8 (±13.8) mm Hg for the control group (*P* = 0.05). The mean diastolic BP at this time point was 71.9 (±7.5) mm Hg for the intervention group, compared with 74.3 (±7.9) mm Hg for the control group (*P* = 0.04).

There was no significant difference in proteinuria or eGFR between the intervention and control groups at baseline nor at any subsequent time point (Supplemental Tables 5A and 5B). Considering all values over the postrandomization period, the mean change from prerandomization eGFR was −3.6 (±13.5) ml/min per 1.73 m^2^ for the control group and 0.3 (±10.2) ml/min per 1.73 m^2^ for the intervention group (*P* = 0.05), with negative values representing a reduction in eGFR over time.

## Discussion

Using an appropriately powered randomized controlled trial in people with CKD, we demonstrate that just 1 month of a simple low-cost intervention resulted in a significant reduction in urine sodium excretion, representing a significant reduction in dietary salt intake. The benefits of this 1-month intervention persisted at a lower level beyond the end of the 1-month intervention during the remaining year of this study.

There is compelling evidence that reducing salt intake delivers health benefits at both individual and population levels.^1–3^ This is especially so in CKD as hypertension is commonly present and renal sodium and water handling may be dysfunctional.^10^ However, despite national and international guidance advising dietary salt reduction in CKD, salt reduction is one of the least achieved targets within guidelines in clinical practice.^19,20^

This likely reflects the difficulty that people with CKD face in trying to reduce their salt intake. Many different types of diet worldwide have become rich in salt, and unlike raw foods, commercially produced or processed foods typically have high levels of salt added to them and contribute substantially to salt intake.^24^ Although some countries have mandatory or voluntary labeling of food salt or sodium content, it can be difficult for people to identify and understand the salt content of commercial foods.^25^ There is also evidence that dietary salt intake is influenced by misconceptions about salt and taste.^26^ This may be particularly problematic as in the early phase after the reduction of dietary salt intake food can taste bland to some people, which may discourage them from persisting with salt reduction.^27,28^

The use of potassium chloride as a salt substitute has been proposed as method of reducing salt intake. A recent study of cluster-randomized rural villages in China used a salt substitute (75% sodium chloride and 25% potassium chloride) or normal salt, and showed a reduction in stroke, major cardiovascular events, and death in people with a history of stroke or who were older than 60 years with high BP.^29^ This study demonstrated the value of reducing salt intake, but unfortunately, a salt substitute cannot address the salt intake arising from ingestion of purchased food that contains high levels of salt. Importantly, potassium chloride supplementation has potential hazards for people with CKD because they are more vulnerable to hyperkalemia.

Clinical trials designed to investigate the health benefits of lowering dietary salt intake have generally used relatively intensive, personalized, and expensive interventions to achieve the reductions necessary to reach statistical significance with relatively small numbers of participants over relatively short periods.^10^ Unfortunately, CKD is common,^30^ and for the most part, such interventions are neither affordable nor feasible for large-scale use, and so cannot be used in routine clinical practice. There is a paucity of evidence about affordable and feasible interventions to help people with CKD to lower their dietary salt intake.

To address this deficit, we tested a simple low-cost care bundle designed to help people with CKD to lower their salt intake. This intervention, the OxSalt care bundle, includes information and real-time reminders and is based on three key guiding principles to help empower people with CKD to lower their dietary salt intake. These three principles are focused on empowering people with CKD to: understand why reducing salt intake is beneficial, understand how to evaluate the salt content of food, and understand how to select or prepare food that is both appetizing and low in salt content. The intervention is based on low-cost digital technology, which can be delivered easily and cheaply at scale, and the provision of some simple printed material. We used routinely available services to host a website, send timed e-mails, and send timed text messages, and the costs for these services were relatively low and would increase very little with scale up of the intervention. The care bundle is a cheap and simple intervention that does not require any individual participant personalization and does not require specialist staff or training to deliver it, so it could easily be integrated into routine clinical practice.

We did not seek to test adherence or engagement with the intervention, but rather to directly test the effects of providing the intervention as this is what could be done in routine clinical practice. The control group received standard routine care and so might have received or self-sourced general advice about salt intake. The purpose of the trial was to test the value of providing the intervention compared with not providing it.

Our study raises the possibility that a longer intervention might result in even more reduction in salt intake. If the effect of the intervention diminishes over time, then there may be value in further, possibly brief, top-up “booster” interventions. Fluctuations in salt intake in the control group might seem to reflect routine care or curiosity about the nature of the intervention that those in the intervention group were receiving, but none of these changes were significant or sustained.

This study was designed and powered to evaluate a change in dietary salt intake as measured using 24-hour urine sodium excretion. Although we measured BP and eGFR, this study was not designed or powered to detect significant changes in these parameters, but a small reduction in diastolic BP was seen with the intervention.

We did not observe any adverse effects from lowering salt intake. Meta-analyses of large population datasets show a J-shaped relationship between salt intake and mortality.^31^ However, because salt is present in most food that is consumed, salt intake can be a surrogate marker for overall food intake. The association of increased mortality with very low sodium intake could represent reverse causation with less well or frailer people having a lower intake of food and so of salt. A detailed study from the Netherlands found increased mortality at low salt intake only in people with a low protein intake and not in people with a healthy protein intake.^32^

Any potential error arising from incomplete 24-hour urine collection should affect the intervention and the control arm equivalently. However, when analyzing the 24-hour urine collections for sodium content, we also measured creatinine content. The calculated sodium:creatinine ratio reduces any potential effect of collection error, and the results of this analysis confirmed the significant sustained reductions in salt intake in the intervention group.

There is evidence of variability of sodium excretion over time within individuals.^33^ However, when comparing the mean values from two groups with similar variance as we have done, with approximately 100 people randomized to each group, such individual variation does not affect the validity of our results, which demonstrate a very clear significant difference in the mean sodium excretion between the control and intervention groups.

The magnitude of the reduction in dietary salt intake of 1.9 g is of real value and notable for an intervention of only 1 month duration. On the basis of estimates by the UK NICE, a smaller reduction in salt intake of 1.4 g/d that occurred between 2003 and 2011 prevented around 9000 cardiovascular deaths per year across the general UK population and saved the UK economy over £1.5 billion per year.^34,35^ The aim of our study was to evaluate whether the intervention worked, and it is promising that even 10 months after the intervention, salt intake was still significantly reduced in the intervention group.

Overall, this randomized controlled study demonstrates conclusively that a short, low-cost intervention, which can be delivered in primary or secondary care, helps people with CKD to lower their salt intake. Future studies are warranted to assess whether further benefit is derived from a longer intervention or later top-up interventions, with the cost reduced even further by large-scale use, which could include online self-enrollment.

## Supplementary Material

## Data Availability

Partial restrictions to the data and/or materials apply: Relevant deidentified data are available on request where this does not risk patient identification.

## Supplemental Material

Supplemental Information 1. The OxSalt Intervention.

Supplemental Figure 1. Sodium excretion over time (mean±SEM) as assessed from 24-hour urine collections.

Supplemental Table 1. Educational Attainment.

Supplemental Table 2. Medication.

Supplemental Table 3. Participants.

Supplemental Table 4. Sodium and Salt excretion.

Supplemental Table 5A. eGFR values.

Supplemental Table 5B. Changes in eGFR values.
