# Supplementary material for: Evaluation of a Simple Low-cost Intervention to Empower People with CKD to Reduce Their Dietary Salt Intake: OxCKD1, a Multicenter Randomized Controlled Trial
Source: Kidney360. 2023 May 31;4(7):890–8. doi: 10.34067/KID.0000000000000160 (PMC10371291; doi:10.34067/KID.0000000000000160)
Supplement: Supplementary file 1 [file kidney360-4-0890-s001.pdf]

Supplementary Material

Supplemental Information 1. The OxSalt Intervention

The intervention is a care bundle designed with the aim of empowering people with CKD to reduce their dietary salt intake. It was developed with input from patients. Three guiding principles underlie the intervention and are focused respectively on empowering people with CKD to:

1. Understand the health benefits of reducing salt intake
2. Understand how to evaluate the salt contents of food
3. Understand how to select or prepare food that is both appetizing and low in salt content

The choice of one month as the duration of the care bundle intervention was pragmatic and based on considerations including consultation and input from patients during the development of the study. A longer intervention would have been more costly and our aim was to test a cheap and simple intervention. We hypothesised that if the intervention worked, then one month would be a long enough time period to allow participants to modify their food purchasing, preparation and consumption to favour foods that were lower in salt and to incorporate any altered behaviour into their regular routine.

Following randomization at the second baseline visit (see Figure 1), the intervention group were provided with a set of brief self-explanatory slides to look over during this visit at their own pace on an tablet or laptop computer; the slides presented key points about salt and provided some relevant examples. The theme of the slides was the three guiding principles above and the pace and timing of their review of the slides was determined by the participants. The purpose of the slides was: to introduce the health benefits of reducing salt intake, to introduce how to evaluate the salt content of food and to introduce approaches to selecting or preparing food that is both appetizing and low in salt content. The following written information was also provided to participants in the intervention group for them to take away in paper form:

- A booklet recapitulating and expanding on the information in the introductory slides—that is, the benefits of reducing salt intake, how to evaluate the salt content of foods and how to select or prepare appetizing food that is low in salt content.
- A small double-sided credit card-sized reminder card about food labelling and salt content of food. This was designed to be usable as a handy reminder when shopping and is reproduced below.
- A booklet containing information and guidance about the salt content of common foods grouped together to help guide decision-making for different meals (e.g. breakfast, lunch, etc.) or snacks.

- A double-sided single-page shopping guide summarizing the salt contents of key common foods and grouping them into high, medium and low salt categories to assist and simplify shopping decision-making. This is reproduced below.
- An information sheet with tips for eating-out to help decision-making around the salt content of foods ordered from a menu.
- An information sheet with simple suggestions for salt-free seasoning for home-cooking.

During the course of the 4-week intervention, participants received a series of automated pre-programmed emails and real-time text messages reminding them about different aspects of the information that they had already been provided with. The text messages and emails were sent out automatically by computer server. Topics in these communications included, for example, motivational messages about the benefits of reducing salt intake, about making decisions about food purchasing and consumption that include consideration of salt content and tips for healthy food choices.

Throughout the 4-week intervention, participants had access to a website containing online versions of the information and guidance that had been provided in the written paper material. The information on the website was the same as the information provided in printed form.

How much Salt?

Use the key below to assess the salt content of different foods

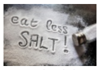

www.studywebsite.org

Salt Per 100g	Salt Per 100g	Salt Per 100g	Salt Per meal
less than 0.3g	between 0.3 and 1.5g	more than 1.5g	between 0.3 and 1.25g

Working out how much salt is in a portion

A food has **0.6g** salt in 100g and the portion size is 300g

300g is 3 x 100g, so the portion contains 3 x **0.6g** salt = **1.8g** salt

A food has 100mg sodium in 100g and the portion size is 300g
(100mg sodium = 0.1g sodium)

0.1g sodium = 2.5 x 0.1g salt = **0.25** g salt
(multipy by 2.5 to convert sodium to salt)

As 300g is 3 x 100g, the portion contains 3 x **0.25g** salt = **0.75g** salt

Low Salt Handy Shopping Guide

Remember that the larger your portion the more salt you are eating

www.studywebsite.org

Limit these foods

They are usually high in salt
Eat only occasionally or as a treat

Bread, cereals & starchy foods

All Bran, Bran Flakes, Corn Flakes, Frosties, Fruit 'n' Fibre, Grapenuts, Oat Bran Flakes, Rice Krispies, Shreddies, Special K, Sultana Bran, Bagel, French stick, crumpets, naan bread, pappadums, instant noodles, tinned spaghetti
Sandwiches made with cheese, processed meat

Meat, Fish & Vegetarian alternatives

Processed meat e.g. bacon, sausages, ham, corned beef, pâté, burgers, faggots, gammon
Meat pie, pasties, sausage rolls, pork pies
Smoked fish, sardines in brine
Processed fish e.g. fish paste, scampi, seafood sticks.
Prawns & shellfish

Eggs & Dairy foods

Most cheeses e.g. cheddar, red Leicester, double Gloucester, cheese spread
Quiche Lorraine, Scotch egg

Vegetables

Hummous

Savoury Snacks

Potato crisps, corn or maize snacks e.g. Wotsits or tortilla chips, prawn crackers, salted popcorn, Twiglets

Sauces & Seasonings

All types of salt e.g. rock, sea & table salt
Soy sauce, Marmite

Check the Label!

These foods contain some salt
Try to choose lower salt options

Bread, cereals & starchy foods

Cheerios, Clusters, Coco Pops, Muesli, Nutri-Grain, Ricicles, Weetabix, Weetos
Bread including malt, rye & soda, pitta bread, chapatti, croissants, English muffins
Cream crackers, water biscuits
Processed potato products e.g. waffles, instant mash, croquettes

Meat, Fish & Vegetarian alternatives

Peanut butter
Offal
Fish in breadcrumbs or batter, fish in sauce, whitebait, tuna in brine, tinned salmon
Quorn

Eggs & Dairy foods

Cottage cheese, cream cheese e.g. Philadelphia
Eggs, plain / cheese omelette
Savoury pancake

Vegetables

Baked beans - reduced salt, Tinned vegetables with added salt, pickled vegetables

Savoury Snacks

Roasted and salted nuts

Sauces & Seasonings

Mayonnaise, tomato ketchup, peanut butter, pasta & curry sauces, packet sauces, Worcestershire sauce, sweet pickle
Oxo cubes, including reduced salt type
Bisto gravy granules, including reduced salt type

Sweet Snacks

Digestive biscuits

Lower Salt Options

These foods are lower in salt
Try to choose these foods most often

Bread, cereals & starchy foods

Muesli with no added salt & sugar, Porridge, Puffed Wheat, Ready Brek, Shredded Wheat, Sugar Puffs
Hot cross buns
Crispbreads e.g. Ryvita, unsalted rice cakes
Grains e.g. couscous, bulgar wheat, quinoa, rice & pasta

Meat, Fish & Vegetarian alternatives

Fresh , lean meat
Fresh white & oily fish
Tinned fish in water, oil or tomato
Tofu

Eggs & Dairy foods

Sweet pancake

Vegetables

Fresh, frozen & dried vegetables & pulses
Tinned tomatoes

Savoury Snacks

Salt 'n' Shake crisps (without the salt sachet), unsalted nuts, plain popcorn

Sauces & Seasonings

Reduced salt tomato ketchup,
HP reduced salt brown sauce, Tomato puree, vinegar, lemon juice, herbs & spices
Heinz for Baby stock cubes,
Kallo very low salt stock cubes

Sweet Snacks

Rich tea biscuits, Gingernut biscuits

Table of Salt Content per portion

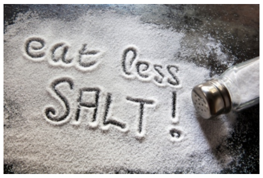

www.studywebsite.org

Salt content in grams per 100g of food

Portion size in grams	**0.1**	**0.2**	**0.3**	**0.4**	**0.5**	**0.6**	**0.7**	**0.8**	**0.9**	**1.0**	**1.1**	**1.2**	**1.3**	**1.4**	**1.5**	**1.6**	**1.7**	**1.8**	**1.9**	**2.0**	**2.1**	**2.2**	**2.3**	**2.4**	**2.5**	**2.6**	**2.7**	**2.8**	**2.9**	**3.0**
**15**	0.0	0.0	0.1	0.1	0.1	0.1	0.1	0.1	0.1	0.2	0.2	0.2	0.2	0.2	0.2	0.2	0.3	0.3	0.3	0.3	0.3	0.3	0.4	0.4	0.4	0.4	0.4	0.4	0.4	0.5
**25**	0.0	0.1	0.1	0.1	0.1	0.2	0.2	0.2	0.2	0.3	0.3	0.3	0.3	0.4	0.4	0.4	0.4	0.5	0.5	0.5	0.5	0.6	0.6	0.6	0.6	0.7	0.7	0.7	0.7	0.8
**50**	0.1	0.1	0.2	0.2	0.3	0.3	0.4	0.4	0.5	0.5	0.6	0.6	0.7	0.7	0.8	0.8	0.9	0.9	1.0	1.0	1.1	1.1	1.2	1.2	1.3	1.3	1.4	1.4	1.5	1.5
**75**	0.1	0.2	0.2	0.3	0.4	0.5	0.5	0.6	0.7	0.8	0.8	0.9	1.0	1.1	1.1	1.2	1.3	1.4	1.4	1.5	1.6	1.7	1.7	1.8	1.9	2.0	2.0	2.1	2.2	2.3
**100**	0.1	0.2	0.3	0.4	0.5	0.6	0.7	0.8	0.9	1.0	1.1	1.2	1.3	1.4	1.5	1.6	1.7	1.8	1.9	2.0	2.1	2.2	2.3	2.4	2.5	2.6	2.7	2.8	2.9	3.0
**125**	0.1	0.3	0.4	0.5	0.6	0.8	0.9	1.0	1.1	1.3	1.4	1.5	1.6	1.8	1.9	2.0	2.1	2.3	2.4	2.5	2.6	2.8	2.9	3.0	3.1	3.3	3.4	3.5	3.6	3.8
**150**	0.2	0.3	0.5	0.6	0.8	0.9	1.1	1.2	1.4	1.5	1.7	1.8	2.0	2.1	2.3	2.4	2.6	2.7	2.9	3.0	3.2	3.3	3.5	3.6	3.8	3.9	4.1	4.2	4.4	4.5
**175**	0.2	0.4	0.5	0.7	0.9	1.1	1.2	1.4	1.6	1.8	1.9	2.1	2.3	2.5	2.6	2.8	3.0	3.2	3.3	3.5	3.7	3.9	4.0	4.2	4.4	4.6	4.7	4.9	5.1	5.3
**200**	0.2	0.4	0.6	0.8	1.0	1.2	1.4	1.6	1.8	2.0	2.2	2.4	2.6	2.8	3.0	3.2	3.4	3.6	3.8	4.0	4.2	4.4	4.6	4.8	5.0	5.2	5.4	5.6	5.8	6.0
**250**	0.3	0.5	0.8	1.0	1.3	1.5	1.8	2.0	2.3	2.5	2.8	3.0	3.3	3.5	3.8	4.0	4.3	4.5	4.8	5.0	5.3	5.5	5.8	6.0	6.3	6.5	6.8	7.0	7.3	7.5
**300**	0.3	0.6	0.9	1.2	1.5	1.8	2.1	2.4	2.7	3.0	3.3	3.6	3.9	4.2	4.5	4.8	5.1	5.4	5.7	6.0	6.3	6.6	6.9	7.2	7.5	7.8	8.1	8.4	8.7	9.0
**350**	0.4	0.7	1.1	1.4	1.8	2.1	2.5	2.8	3.2	3.5	3.9	4.2	4.6	4.9	5.3	5.6	6.0	6.3	6.7	7.0	7.4	7.7	8.1	8.4	8.8	9.1	9.5	9.8	10.	10.
**400**	0.4	0.8	1.2	1.6	2.0	2.4	2.8	3.2	3.6	4.0	4.4	4.8	5.2	5.6	6.0	6.4	6.8	7.2	7.6	8.0	8.4	8.8	9.2	9.6	10.	10.	10.	11.2	11.6	12.

Supplementary Figure 1

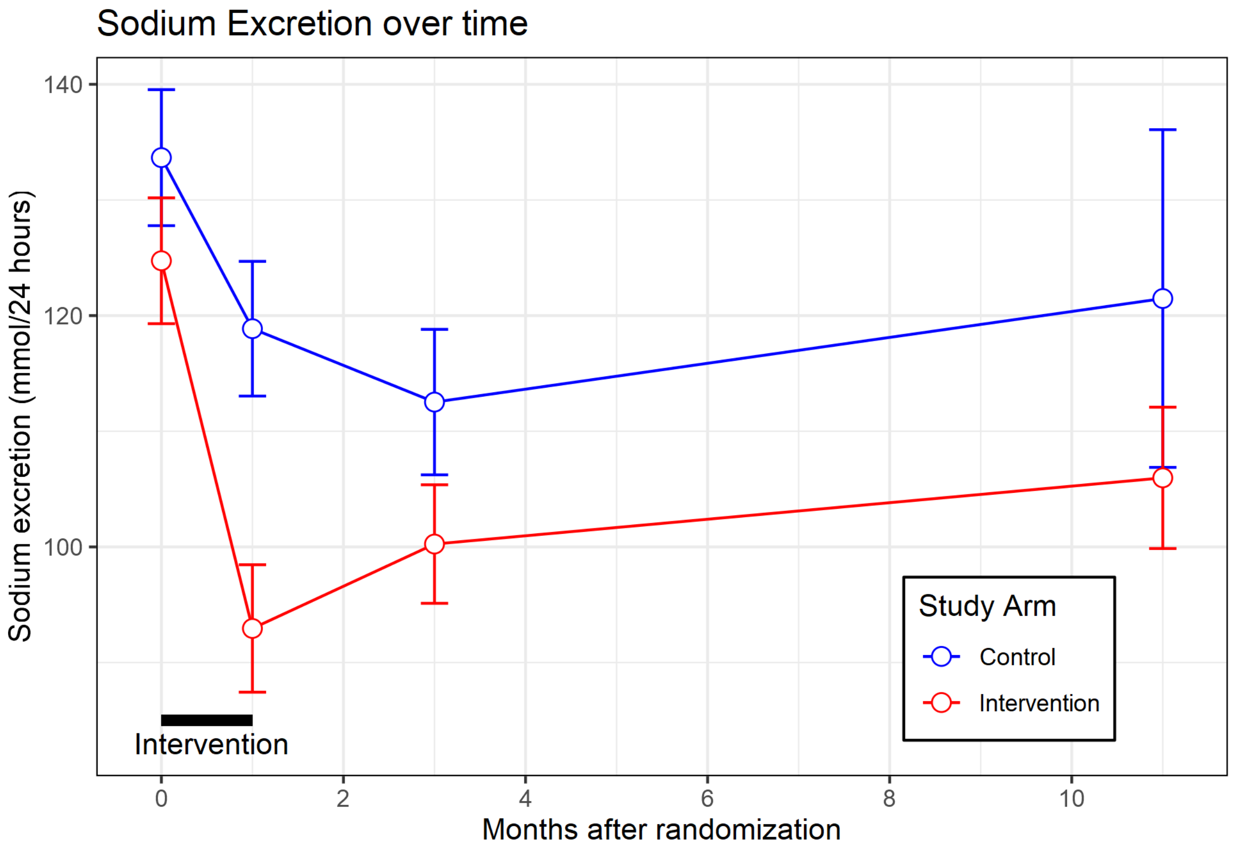

Supplementary Figure 1. Sodium excretion over time (mean ± standard error) as assessed from 24-hour urine collections.

Supplementary Table 1

Supplementary Table 1: Educational Attainment

Self-reported highest educational attainment of participants who provided this information

	Control	Intervention
Finished school at 16 or younger without taking any qualifications	34	26
Finished school at 16 with one or more qualifications	11	15
Vocational training or apprenticeship	19	13
Finished school age 18 with one or more higher qualifications taken	9	8
Finished university or other higher education with a degree or diploma	23	33

The control and intervention groups do not differ significantly (chi-square, $p = 0.33$)

Supplementary Table 2

Supplementary Table 2: Medication

Drugs prescribed for participants

	Group		
**Drug**	**Control**[1]	**Intervention**[1]	**p-value**[2]
ACEI	40 (41%)	42 (44%)	0.8
ARB	18 (19%)	9 (9.5%)	0.10
ACEI or ARB	56 (58%)	51 (54%)	0.7
Furosemide	6 (6.2%)	10 (11%)	0.3
Thiazide	12 (12%)	9 (9.5%)	0.6
Any diuretic	18 (19%)	18 (19%)	>0.9
Bicarbonate	2 (2.1%)	3 (3.2%)	0.7

ACEI, angiotensin converting enzyme inhibitor; ARB, angiotensin receptor blocker

[1]n (%)

[2]Fisher's exact test

Supplementary Table 3

Supplementary Table 3: Participants

Numbers providing 24-hour urine collections

	Control Group	Intervention Group
1 month	81	77
3 months	69	65
11 months	16	44

Time periods are from the start of the 1-month intervention.

Supplementary Table 4

Supplementary Table 4: Sodium and Salt excretion

24-hour urine collections

	Sodium (mmol/day)		Salt (grams/day)	
**Timepoint**	**Control**[1]	**Intervention**[1]	**Control**[1]	**Intervention**[1]
First Baseline	130 (60)	130 (60)	7.6 (3.5)	7.6 (3.5)
Second Baseline	132 (57)	118 (56)	7.7 (3.4)	6.9 (3.3)
Mean Baseline	134 (58)	125 (53)	7.8 (3.4)	7.3 (3.1)
1 month	119 (52)	93 (48)	6.9 (3.1)	5.4 (2.8)
3 months	113 (52)	100 (41)	6.6 (3.1)	5.9 (2.4)
11 months	121 (58)	106 (41)	7.1 (3.4)	6.2 (2.4)

The mean baseline represents the mean of the first and second baseline measurements.

[1]Mean (SD)

Supplementary Table 5A

Supplementary Table 5A: eGFR values

**Timepoint**	**Control**[1]	**Intervention**[1]	**p-value**[2]
First Baseline	70.4 (36.1)	61.0 (27.9)	0.10
Second Baseline	70.7 (38.8)	64.2 (28.2)	0.5
Mean Baseline	68.7 (34.4)	60.0 (25.8)	0.082
1 month	58.6 (26.0)	68.7 (31.7)	0.2
3 months	65.6 (33.5)	63.7 (30.2)	0.8
11 months	70.2 (42.9)	54.8 (27.7)	0.3

Time periods are from the start of the 1-month intervention. The mean baseline represents the mean of the first and second baseline measurements. eGFRs in ml/min/1.73m2

[1]Mean (SD)

[2]Welch Two Sample t-test

Supplementary Table 5B

Supplementary Table 5B: Changes in eGFR values

Changes from mean baseline

**Timepoint**	**Control**[1]	**Intervention**[1]	**p-value**[2]
1 month	-1.9 (12.7)	0.7 (7.0)	0.5
3 Months	-5.5 (13.2)	1.0 (12.6)	0.11
11 Months	-2.0 (15.9)	-0.9 (11.0)	0.8

Time periods are from randomization at the start of the 1-month intervention. eGFRs in ml/min/1.73m2

[1]Mean (SD)

[2]Welch Two Sample t-test
